# Biomarkers of Environmental Toxicants: Exposure and Biological Effects

**DOI:** 10.3390/toxics8020037

**Published:** 2020-05-22

**Authors:** Robert J. Turesky, Kun Lu

**Affiliations:** 1 Masonic Cancer Center and Department of Medicinal Chemistry, University of Minnesota, 2231 6th St. SE, Minneapolis, MN 55455, USA; 2 Department of Environmental Sciences and Engineering, University of North Carolina at Chapel Hill, Chapel Hill, NC 27599, USA

Biomarkers of environmental toxicants are measures of exposures and effects, some of which can serve to assess disease risk and interindividual susceptibilities. Metabolites, protein, and DNA adducts also serve to elucidate mechanisms of bioactivation and detoxication of reactive toxicant intermediates. Some environmental chemicals act as modulators of gene and protein activity and induce dysbiosis of the microbiome, which impacts the metabolome and overall health. In this Special Issue on “Biomarkers of Environmental Toxicants”, original research and review articles are reported on the latest biochemical, bioanalytical, and mass spectrometry-based technologies to monitor exposures through targeted and nontargeted methods, and on mechanistic studies that examine the biological effects of environmental toxicants in cells and humans. 

The exposome, or the totality of environmental exposures, represents both internal exposures originating from host physiology and external exposures deriving from diverse toxicants and chemicals, infectious agents, as well as diet and drugs. Exposome has been proposed to be a critical entity of human disease. Xue et al. reviewed the current chemical exposome measurement approaches with a focus on those based on the mass spectrometry [1]. They further explored the strategies in implementing the concept of chemical exposome and discussed the available chemical exposome studies. Early progress in chemical exposome research was outlined, and major challenges were highlighted. Apparently, efforts towards chemical exposome have only uncovered the tip of the iceberg, and further advancement in measurement techniques, computational tools, high-throughput data analysis, and standardization would allow for more exciting discoveries to be made concerning the role of exposome in human health and disease.

The gut microbiome has emerged as a new mediator in human health. On the other hand, the human gut microbiome can be easily disturbed upon exposure to a range of toxic environmental agents. Environmentally induced perturbation in the gut microbiome is strongly associated with human disease risk. Functional gut microbiome alterations that may adversely influence human health is an increasingly appreciated mechanism by which environmental chemicals exert their toxic effects. Tu et al. defined the functional damage driven by environmental exposure in the gut microbiome as gut microbiome toxicity [2]. The establishment of gut microbiome toxicity links the toxic effects of various environmental agents and microbiota-associated diseases, calling for a more comprehensive toxicity evaluation with an extended consideration of gut microbiome toxicity.

Living organisms respond to environmental changes and xenobiotic exposures via diverse mechanisms. tRNA-mediated mechanisms are only recently emerging as important modulators of cellular stress responses. Huber et al. discussed many ways that nucleoside modifications confer high functional diversity to tRNAs, with a focus on tRNA modification-mediated regulation of the eukaryotic response to environmental stress and toxicant exposures [3]. Additionally, the potential applications of tRNA modification biology in the development of early biomarkers of pathology are also highlighted. Their review highlights a high functional diversity, ranging from the control of tRNA maturation and translation initiation, to translational enhancement through modification-mediated codon-biased translation of mRNAs encoding stress response proteins, and translational repression by stress-induced tRNA fragments. Future work in this area would provide more exciting discoveries to better understand the role of tRNA modification in exposure-induced human disease.

Exposure to heavy metals is ubiquitous and has been associated with a number of human diseases. Biomarkers of heavy metal exposure in children are particularly important for monitoring exposure and health risk assessment. The study by Jursa et al. measured hair Mn, Pb, Cd, and As levels in children from the Mid-Ohio Valley to determine within and between-subject predictors of hair metal levels. Specifically, occipital scalp hair was collected in 2009–2010 from 222 children aged 6–12 years (169 females, 53 males) participating in a study of chemical exposure and neurodevelopment in an industrial region of the Mid-Ohio Valley [4]. They found that hair Mn and Pb levels were comparable (median 0.11 and 0.15 µg/g, respectively) and were ~10-fold higher than hair Cd and As levels (0.007 and 0.018 µg/g, respectively). In addition, metal levels were different between male and female subjects and showed different profiles along hair segments.

Benzene is a known carcinogen and causes hematotoxicity. Benzene exposure occurs through factory occupations, and from emissions of burning coal and oil in the air. Cigarette smoking is another important source of exposure to benzene. In this research article, Tranfo and colleagues employed a targeted LC-MS/MS to biomonitor benzene through the biomarker S-phenyl-mercapturic acid (SPMA), which is formed from the benzene oxide metabolite conjugated with glutathione (GSH), and then excreted in urine as SPMA [5]. The findings reveal that the main source of benzene exposure in a cohort not occupationally exposed to benzene in central Italy was through active smoking; however, nonsmokers were also exposed to airborne concentrations of this carcinogen.

Phthalates are used as plasticizers and additives in many consumer products. Human exposure to phthalates is prevalent and occurs mainly through dietary sources, dermal absorption, and inhalation. Laboratory animal studies reveal endocrine-disrupting and reproductive effects of phthalates. Thus, human exposure to phthalates is a public health concern. Wang and colleagues have compiled a review on the biomonitoring studies of phthalates in populations across the globe and associated adverse health effects. Urine, serum, amniotic fluid, breast milk, semen, and saliva serve as biospecimens to screen phthalate metabolites. Epidemiological studies have linked high exposure to phthalates with sex anomalies, endometriosis, altered reproductive development, early puberty and fertility, breast and skin cancer, allergy and asthma, overweight and obesity, insulin resistance, and type II diabetes [6]. 

Aldehydes are ubiquitous in the environment, originating from man-made sources, tobacco smoke, and natural processes. Some aldehydes are implicated in diseases, including diabetes, cardiovascular diseases, neurodegenerative disorders (i.e., Alzheimer’s and Parkinson’s Diseases), and cancer. Aldehydes are strong electrophiles that react with nucleophilic sites in DNA and proteins to form reversible and irreversible modifications. These modifications, if not eliminated or repaired, can lead to alteration in cellular homeostasis, cell death, and contribute to disease pathogenesis. In this review, Dator and colleagues describe the metabolism of aldehydes in vivo, and the bioanalytical, and mass spectrometry-based approaches to characterize aldehydes in cells and biomonitoring in humans [7].

Hemoglobin (Hb) and albumin (Alb) are the most abundant proteins in the blood and form covalent adducts with toxicants and endogenous electrophiles. In this review, Preston and Phillips describe targeted and nontargeted mass spectrometry-based strategies to measure exposures to a wide range of toxicants that alkylate the N-terminal valine residues of the α-chain of Hb, and the cysteine residue (Cys-β93) of the β-chain of Hb, which reacts with many electrophiles, including carcinogenic aromatic amines [8]. The histidine residues of Alb react with epoxides of polycyclic aromatic hydrocarbons, and lysine residues form adducts with aflatoxin B_1_ dialdehyde. The highly nucleophilic Cys-34 residue of Alb is the only site for which untargeted adductomic methods have served to screen for electrophiles in humans. Protein adductomics techniques can screen for harmful exposures to causative agents of chronic disease and identifying individuals at risk.

In this research article, Aasa and colleagues employed the *N*-(2,3-dihydroxypropyl)valine hemoglobin adduct formed with glycidol, a carcinogen present in refined edible oils, to assess internal doses of this genotoxicant in a cohort of children [9]. The adduct showed a fivefold variation between the children. The estimated mean intake of glycidol (1.4 μg/kg/day) was about two times higher than the estimated intake for children by the European Food Safety Authority. The estimated lifetime cancer risk (200/10^5^) was calculated by a multiplicative risk model from the lifetime in vivo doses of glycidol in the children and exceeded the acceptable cancer risk estimate. The protein adduct biomarker data, calculated intakes, and corresponding estimated cancer risks emphasize the importance of identifying and mitigating the sources of background exposure to glycidol from foods and other possible sources.

Exposure to environmental chemicals often leads to diverse DNA damage, and the formation of DNA adducts is one of the key events in chemical-induced carcinogenesis. It is critical to determine whether DNA adducts cause mutagenesis. Chemical incorporation of a modification at a specific site within a vector (site-specific mutagenesis) has been a useful tool to deconvolute what types of damage quantified in biologically relevant systems may lead to toxicity and/or mutagenicity, thereby allowing researchers to focus on the most relevant biomarkers that may impact human health. Here, Bian et al. introduced shuttle vector-based methods and reviewed a sampling of the DNA modifications that have been studied by shuttle vector techniques [10]. 

A significant limitation in biomonitoring cancer-causing agents is the paucity of fresh frozen tissues available for DNA adduct biomarker research. In this review, Yun and colleagues report on the methods commonly used to biomonitor DNA adducts, and the use of formalin-fixed paraffin-embedded (FFPE) tissues for the measurements of DNA adducts of genotoxicants found in the diet and tobacco smoke [11]. The authors developed a technique to retrieve the DNA adducts from FFPE tissues under mild conditions that completely reverses the DNA crosslinks while preserving the structures of the DNA lesions. FFPE tissues for which there is a clinical diagnosis of disease present a previously untapped source of biospecimens for molecular epidemiology studies that seeks to assess the causal role of environmental chemicals in cancer etiology. 

DNA adducts are believed to play a central role in the induction of cancer in cigarette smokers. Ma and colleagues have summarized the research on DNA adducts formed with carcinogens in tobacco smoke and from oxidative DNA damage [12]. The analytical approaches most commonly used are mass spectrometry (MS), ^32^P-postlabeling, and immunohistochemistry. Because of the high selectivity and sensitivity, MS methods are the preferred technique and have largely supplanted immunochemical and postlabeling techniques over the past decade. DNA adducts of different classes of tobacco carcinogens have been identified in human biospecimens. Issues pertaining to the validation of DNA adducts such as biomarkers, mitigation of artifacts, and caveats in the designs of human studies are highlighted. 

Aristolochic acids (AAs) are found in *Aristolochia* plants, some of which have been used in the preparation of traditional herbal medicines worldwide. AAs are highly nephrotoxic and carcinogenic to humans and implicated as causative agents of the Balkan endemic nephropathy (BEN) and “Chinese herbs nephropathy” in Asia. In this article, Chan and colleagues provide an overview of the exposure of AAs in the Balkan Peninsula, where the comingling of *Aristolochia* plants with grains and the release of AAs from decayed seeds of *Aristolochia* plants contaminate the agricultural soil, the food crops, and the water supply [13]. The links between exposure to AAs and their biomarkers of DNA damage, mutations in cancer driver genes, and mechanisms of kidney fibrosis in Asian cohorts are reported. 

Accelerator mass spectrometry (AMS) is an exquisitely sensitive technique to measure long-lived radionuclides that occur naturally in the environment. AMS has been used for many years in the earth sciences, such as for radiocarbon dating in archaeology. In this review, Malfatti and colleagues describe the approaches and advances employing AMS in human health and risk assessment. The applications of radiocarbon tracer technology in cancer-related studies assessing low-dose toxicology studies of naphthalene-DNA adduct formation, benzo[a]pyrene pharmacokinetics in humans, and the antibacterial triclocarban exposure and impact on the endocrine system are reported [14]. AMS applications in precision medicine include the use of radiocarbon-labeled cells for better defining mechanisms of metastasis and the use of drug-DNA adducts in the in vivo and ex vivo microdosing strategy of chemotherapeutics as predictive biomarkers of interindividual response to chemotherapy.

In summary, this collection of original research and review articles provides a valuable update of the most recent biochemical and analytical tools that employ biomarkers in toxicology research, biomarker discovery, and exposure and risk assessment in population-based studies. 
